# Residents’ experiences of paternalism in nursing homes

**DOI:** 10.1177/09697330231166085

**Published:** 2023-07-31

**Authors:** Anne Helene Mortensen, Dagfinn Nåden, Dag Karterud, Vibeke Lohne

**Affiliations:** Faculty of Health, Department of Nursing and Health Promotion, 158935 Oslo Metropolitan University, Oslo, Norway

**Keywords:** Paternalism, autonomy, dignity, long-term care facilities, qualitative research

## Abstract

**Background:**

Interest in strengthening residents’ autonomy in nursing homes is intensifying and professional caregivers’ experience ethical dilemmas when the principles of beneficence and autonomy conflict. This increased focus requires expanded knowledge of how residents experience decision-making in nursing homes and how being subject to paternalism affects residents’ dignity.

**Research question/aim:**

This study explored how residents experience paternalism in nursing homes.

**Research design:**

This study involved a qualitative interpretive design with participant observations and semi-structured interviews. The interpretations were informed by Gadamer’s hermeneutics.

**Participants and research context:**

Eleven residents were interviewed after a period of participant observation in two nursing homes.

**Ethical considerations:**

The study was performed in accordance with the Helsinki declaration. The Regional Ethics Committee (REK) permitted the researcher to perform participant observation in the nursing homes. The use of audio recordings of interviews was registered and supervised by Sikt - Norwegian Agency for Shared Services in Education and Research. The resident’s consent was assessed continuously. Three interviews were terminated for ethical reasons.

**Findings:**

The resident interviews revealed that residents found it obvious for caregivers to possess the decision-making authority in nursing homes. When residents explained their views, three main themes emerged: (1) To be included even though caregivers make the decisions, (2) Surrender to dependency, and (3) Adherence to nursing home norms.

**Conclusions:**

Residents submit to their caregivers and give caregivers the responsibility and function as leaders. Paternalism was experienced as dignifying in situations where it contributed to residents being able to live according to second order desires and values, and when it implied respect and appraisal of residents’ capabilities. Paternalism was experienced as debasing when residents felt left out, and when residents felt that their capabilities were underestimated. This also included their capability to withstand paternalistic influence.

## Introduction

Historically, health care services have been characterized by paternalism, with health professionals having more power than patients and residents.^1^ The last 30 years, there has been an increasing focus on strengthening residents’ autonomy to preserve their dignity in nursing homes, based on dignity as an important ethical value.^2,3^ However, professional caregivers in nursing homes face ethical dilemmas when the principles of beneficence and autonomy conflict,^4^ and the resident autonomy is sometimes cited as a reason for not providing them with the necessary care.^3,4^ The core tension within discussions about autonomy and beneficence in health care is paternalism.^5,6^ Considerable research has been devoted to understanding residents’ need for autonomy^2,3^ while less attention has been paid to their experiences with paternalism. Therefore, we need more and new knowledge about how residents experience decision-making and paternalism in nursing homes

## Background

The absolute ethical is found in our acceptance of the absolute dignity of man, of his inviolability in all respects.^7^ Hence, the preservation of dignity is a primary ethical goal in health service and policy.^8–10^ Nursing homes are known to be sites where dignitarian norms can become difficult to uphold,^11^ and where residents are sometimes subject to disrespectful treatment.^8^ Previous research found that stigma, objectification, haste, and misunderstandings contribute to unworthy care in nursing homes, and that residents’ vulnerability to undignified care is exacerbated by a lack of reciprocity in the care relationship, including a reduced ability to communicate.^12^ Similarly, residents’ autonomy diminishes with increased vulnerability and dependency.^13^

Residents perceive autonomy as the opportunity to make their own decisions, this decision-making power is related to their educational level and physical, mental, and financial capacity. In contrast, increased dependence increases the risk of being exposed to paternalistic behaviour.^2^ Professional caregivers, relatives, care practices, and a residential care environment can support and/or hinder residents’ autonomy.^3^ Caregivers in nursing homes support residents’ autonomy by acting as their advocates, respecting their wishes and protecting their rights to make decisions, by fostering independence, sharing information, and by providing individualised care.^3^ Residents perceive support for autonomy as a sign of respect and dignity, while hindrance of autonomy leads to feelings of confinement and frustration.^2^ Within care practices, there is a need to balance several components – resident’s wishes against relatives’ wishes, scarcity of resources, an assumption that nursing home residents are not able to make autonomous decisions – these are barriers to maintaining residents autonomy, causing caregivers to make decisions for the resident with the aim of protecting them. However, resident’s autonomy is sometimes also used as justification for failing to provide residents with necessary care.^3^

Considerable research has been devoted to understanding residents’ experience of autonomy and dignity,^2,3,8,12,14^ with less attention being devoted their experiences of paternalism.

## Aim

This study aims to explore how residents experience paternalism in nursing homes.

## Theoretical framework

Dignity has both an objective and a subjective dimension. Objective dignity is the basis of human rights, where it is seen as a ‘value’ that a person possesses simply by being a human being. Subjective dignity includes the idea that individual differences can be considered owing to one’s experiences.^7, 9–11,15,16^ In this study, dignity is understood as consisting of intertwined strands of personal dignity, social dignity, and status dignity. Residents have and experience personal and social dignity in the way dignitarian norms, which relate to them, are upheld or transgressed. Transgression of dignitarian norms entails withheld esteem extending to disdain and shame. Status dignity calls on others to treat us with the respect appropriate for our status or position (human being, doctor, resident, etc.). Status dignity cannot be reduced, like personal and social dignity, but is violated when individuals are not treated with the respect that their status commands.^11^

The concept of autonomy refers to the rights to self-government; the right to make our own decisions, decide our own values and ends, and pursue these.^6^ The concept also describes conditions used to determine if a choice is autonomous or not.^6^ In our understanding, an autonomous choice must be motivated by second order values and desires, it cannot be the product of oppression or manipulation and it should be justifiable by the one making the choice. Furthermore, the concept of autonomy is used to describe the capacity for autonomy, which requires rationality and critical reflection.^6,17^

Acting against someone’s explicit will is considered paternalism.^6^ In strong paternalism the protection of the resident’s wellbeing is the sole goal. If the resident’s behaviour is not truly autonomous or their capacity for autonomy is compromised, we consider it soft paternalism.^6,17,18^ In addition, soft paternalism aims to bring the persons choices and actions in line with the persons second order desires or true values. Thus, soft paternalism presupposes a mismatch between the expressed will and the authentic will.^6^ We can also differentiate between soft and strong paternalism, based on how easy the influence is to resist. Hence, influence which is supposed to be resistible, is considered soft paternalism,^19^ whereas coercion, which is influence that is harder or impossible to resist, is considered strong paternalism.

## Research design

This study has a qualitative design, informed by Gadamer’s hermeneutics. This approach emphasizes the researcher’s preconceptions and their recognition of the same.^20^ Preconceptions in this study were influenced by professional caregivers’ experience of dilemmas between residents’ right to autonomy and their duty to uphold the principles of beneficence,^4^ concepts of dignity, and paternalism.^6,21,22^ Data were collected using semi-structured qualitative interviews, after a period (7/8 weeks) of participant observation at two different nursing homes.

## Participants and context

The two nursing homes were chosen purposely to ensure variance regarding access to resources and experience linked to the use of paternalism and coercion. A total of 11 residents were interviewed. The inclusion criteria for participation in interviews were residents in a participating nursing home and being able to give informed consent. A total of 80% of nursing home residents in Norway suffer from dementia.^23^ Therefore, we did not distinguish between residents with and without dementia. The sample was chosen purposely, striving for a balanced gender representation, variance in physical and mental function, and history of acceptance and/or resistance to care (Table 1). Registered nurses involved in daily care and with extensive knowledge of the resident, recruited residents for participation. Selection of informants was made towards the end of the participant observation period, to establish trust and reach consensus concerning the inclusion criteria.

**Table 1.** Participants’ characteristics^a^ and duration of the audio recordings.

Nursing home	Resident	Gender	Age (years)	Minutes^b^
Nursing home 1				
	R1	Female	98	29
	R2	Female	87	5^c^
	R3	Male	91	18
	R4	Male	98	44
	R5	Female	94	75
	R6	Female	91	30
Nursing home 2				
	R7	Female	84	19
	R8	Male	89	44
	R9	Female	90	21 + 4
	R10	Male	86	16
	R11	Male	81	25

^a^Physical and mental function, and history of acceptance and/or resistance to care is not included to ensure resident confidentiality.

^b^Introductions, information about the study, and re-assessments of the resident’s consent were not audio-recorded.

^c^The interview was interrupted after 5 minutes.

## Data collection, analysis, and interpretation

All interviews were conducted in the privacy of the residents’ rooms, and they were ensured confidentiality. The residents and researcher conducting the interviews were somewhat familiar with each other, through a participant observation preceding the interviews. The residents knew the researcher’s background and were informed that she wanted to learn about life in the nursing home. The residents were encouraged to talk about how they experienced living in the nursing home, cooperation with those working there, who was making decisions, and if there were things that they did not like. Follow-up questions and silence were used to stimulate residents to elaborate on their descriptions. Paraphrasing was used to verify the interviewer’s first interpretations.^24^ The interviews were rounded off by asking residents if there were other things they wanted the researcher to know. Field notes were written immediately after each interview. The audio recordings were continuously uploaded to a secure platform and transcribed verbatim by the interviewer (first author). Quotes were translated and edited for clarity.

Interpretation started during data collection, wherein preunderstanding and the study’s aim influenced what the researcher heard and interpreted.^20^ The interpretations made during the interviews influenced follow-up questions.^24^ The study’s aim and preunderstanding were crucial for the authors’ understanding.^20^ After these initial steps of interpretation, the first author carried out a formal analysis through an iterative process of coding and interpretation, described by Brinkmann and Kvale ^24^ as a bricolage analysis. The first step of the analysis was open reading to get to know the material.^24^ In this phase, field notes were read to support and validate interpretations. During the open reading, the researcher wrote memos with preliminary ideas.^24^ The first coding of the interviews was an inductive open coding of manifest units of meaning. The analysis and interpretation of the interviews continued in an iterative movement between data extracts (codes), interpretations (themes), and the whole data corpus.^20^ NVivo was used to organize the material and keep track of data extracts and interpretations. The first steps in the analysis were completed by the first author (AHM) under supervision of the last author (VL). Towards the end of the analysis, the other authors (DN and DK) were included, verifying themes and interpretations. The interpretations considered three contexts of understanding: the informant’s self-understanding, a critical common-sense understanding, and a theoretical understanding (Table 2).^24^

**Table 2.** Examples of data and levels of interpretation for the theme ‘adherence to nursing home norms’.

Data	Self-understanding	Common-sense understanding	Theoretical understanding
P4: I would have gone crazy if I worked here. (……) There is this one (resident) who at every meal drinks up her milk, and then she puts the glass in the bag. She takes it with her, and there is a married couple here, they are like cats and dogs, always fighting…	Caregivers have a difficult job	There are defined norms in the nursing home. These norms say that you shall be modest and grateful, you shall not complain, be a burden, or demand more	Breaking of the norms leads to condemnation and informal sanctions. Signs of reduced personal and social dignity
	Other residents are demanding, they are not behaving properly		
(…..) I think I am on relatively good terms with the staff here, because I very rarely call on them, and I burden them as little as possible, and I say thank you. I am not the one who plunges forward, I think. I try to cooperate	To be a good resident		
	Not be a burden		
	Be grateful		
	Be modest and cooperative		
P5: I went to the doctor for a check-up of my pacemaker once, and they did not know about it here, and when I came back, they came like a lynching mob (…..)	Reprimanded for seeing the doctor	Failure to comply to the norms implies condemnation and informal sanctions	Compliance to the norms leads to the experience of personal and social dignity
It seems that they are afraid that one will get a little more than the others here. (…..) I felt so stupid (……). Now, when I come in and say, ‘Good morning, everyone!’, no one answers, and I wonder if I said something wrong	Taking up resources		
	Being ignored		
	Feels like she did something wrong		
P8: (….) but it is a little difficult. I need help for that, so I prefer to… it is good food and there is nothing to really complain about here (…..) I do understand that they have a lot of other chores too. And I do not want to disturb them if possible	Hesitant to ask for help	‘A good resident’, adherence to nursing home norms	The nursing home norms are dignitarian norms
	Will not complain		
	Do not want to disturb or be a burden		

## Ethical considerations

The principles of informed consent, confidentiality, and assessment of consequences for participants were observed throughout the entire study. The regional Ethics Committee (REK) permitted the researcher to perform participant observation in the nursing homes. The use of audio recordings was supervised and registered by Sikt -Norvegian Agency for Shared Services in Education and Research. All residents received written and oral information, were assured confidentiality, and informed that they could withdraw from the study at any time.

When there was doubt regarding a resident’s competence to consent, informed consent was obtained from both the resident and their guardian. Nurses who knew the residents assisted in this assessment. The researcher continually reassessed residents’ consent before and during the interviews.^25^ Three interviews were terminated, and all information including consent forms were deleted, when residents’ showed signs of distress, or did not understand the consent.

## Findings

The analysis and interpretation of the interviews revealed that the residents thought it was obvious that caregivers had the decision-making authority in the nursing home. When residents explained their views, three main themes emerged; (1) To be included even though the caregivers make the decisions; (2) Surrender to dependency; and (3) Adherence to the nursing home norms.

### To be included even though the caregivers make the decisions

The residents expressed that it was mostly the caregivers who made decisions in the nursing home. Some of the residents said that they had ‘nothing to say in the nursing home’, whereas most of them expressed that even though the caregivers made most of the decisions, they could ‘express wishes’ and the caregivers would listen. If something was important to them, the caregivers would take those wishes into consideration.

P11: I can say no. I can do that. They respect that. So do the physiotherapists. Yes, when they push me, and when my leg gives in, I say no, and then they respect it.

Residents also told the interviewer that the caregivers often urged them to express their wishes and let them know what they needed, but the residents were reluctant to do so.

P4: (....) They (the caregivers) say ‘you just give a signal if there is something you need, I will come and help you’, but I am very wary to do that.

Although the residents were reluctant to express wishes and needs, it seemed important for the residents that they were given the opportunity to do so.

Major decisions regarding their future and/or health issues were made either by the caregivers alone, or by a team consisting of professional caregivers, the resident, and their relatives. Some residents expressed that they were actively involved in these decisions. Others stated that these decisions were mainly made by professional caregivers and relatives. The experience of being included depended on whether the residents received sufficient information throughout the decision-making process. One resident had just experienced that her next of kin and professional caregivers decided about her stay in the nursing home rehabilitation unit, without her knowledge.

P9: No one has told me that I am going to be here for so long. They have told my children, but not me. I think that is awful.

I: Did you participate in the decision?

P9: No, not me. I think my kids decided that.

I: Do you agree with the decision?

P9: Yes, it makes sense. Because I do not have family living at home anymore… so it does….. The children have to help me, and they all work…. and I cannot manage being on my own anymore either….

This resident expressed severe discomfort because she had been left out of the decision, even though she agreed with the reasoning and result. What seemed to make the situation debasing to her was that she did not even know that an extension of the stay was being considered, including the experience of being the last one to know.

Being included and having the opportunity to express their wishes seemed more important than being the one who made the decision or agreement with the decision.

### Surrendering to dependency

The residents expressed that it was difficult to accept that they could no longer manage on their own at home; they could not be independent and self-sufficient and needed more help than the family could provide. They expressed that in this situation, they felt it was natural to surrender the control to those helping them.

P3: I have left (surrendered) myself to this, to being here, so they decide. At least now that I am so unwell, I do not have anything to say. It is they (the caregivers) who know about this.

Residents also described being dependent on the caregivers for motivation. Some residents described a general lack of motivation; other residents described how pressure from the caregivers was necessary for them to keep doing things themselves, to preserve physical and social function, participate in activities, uphold standards of hygiene, or accomplish rehabilitation goals.

P9: I say ‘no’, and then they say, ‘You can do a little more’ and then there is nothing to do, but to just do it, and then I mostly manage it. That there is someone who does not give up until I really must give up, that is important.

Residents expressed that the professional caregivers were the ones who possessed the knowledge, and it was natural to do as the ‘experts’ say. Only when residents felt unsure whether the caregivers had enough knowledge, or if they felt that the caregivers did not understand them or the complexity of their situation, did they express discomfort related to surrendering control to the caregivers. For instance, there was this resident who was living with a chronic ulcer. He had tended to his ulcer himself for many years before moving to the nursing home.

P8: Sometimes I have to tell them how to do it, and then they do it. Some (caregivers) are very good, others .... I do not know how long their education is and stuff, but this is not a wound unit, you know…

I: Do you feel that you have more control when you take care of it yourself then?

P8: Yes, yes. Yes exactly. The wound, I want to take care of the wound myself.

For the residents in the rehabilitation unit, this surrender of control to the caregivers seemed related to a hope of getting better, and of being able to manage at home again after the stay. Whereas residents in the long-term unit seemed to be more concerned with accepting the situation and settling down.

### Adherence to nursing home norms

The residents experienced the nursing home as a larger social unit where they had to fit in. It seemed obvious to them that they should comply with the nursing home’s routines as well as the caregivers’ different ways of doing things.

P4: I have not met the top management here, but I reckon that when it (the nursing home) has been in operation for 10–20 years, they have had so many years to adjust things; so, I should be very careful about giving them any advice on how to run things.

The common areas of the nursing home were not considered ‘home’ in the sense of a place where you could be private and yourself. Only the residents’ rooms were referred to as private, and a place where they could be ‘free’.

P10: What I think is nice here, is that I have this room. Here (in my room) I can be myself and I can be alone and just do as I want to.

Throughout the interviews, we identified several norms regulating the residents’ behaviour. The norms that seemed especially important were those that prescribed residents to accept the situation, be modest and appreciative, and be grateful for receiving help. Sometimes, the norms were directly expressed by residents who declared: ‘I am grateful for help’, and ‘she says I have to learn to accept it, but I cannot’. Residents conformed to these norms, wanting to be ‘good residents'.

P4: I think I am on relatively good terms with the staff here, because I very rarely call on them, and I burden them as little as possible.

Failure to conform to the norms implied condemnation, sometimes extending to informal sanctions. This resident felt that she was reprimanded and ignored for breaking nursing home norms.

P5: But now I think people….. When I come in and say, ‘Good morning, everyone’, No one answers, and I wonder if I said something wrong,

Compliance to the norms and routines were not considered all negative by the residents. Residents also brought up examples of how routines could be a good thing that helped them.

P8: Here, it is more structure and order, and I really benefit from that. There were so many things I could have been involved in at home… social happenings for the elderly… I could have been involved in those, but then it often started at 11.00 a.m. and at that time, I had not even had my breakfast yet.

This resident experienced that the routines of the nursing home helped him structure his life, participate in activities, and be social, doing all the things that he appreciated. Other residents reported that they had struggled to maintain regular meals and activities. ‘Things had started to slip’ at home.

Transition to the nursing home implied a big change for the residents. They transitioned from being alone or in a very small familiar social context, to a new larger social context, with less privacy, new norms, rules, and ways of doing things.

## Discussion

Our findings show that residents experience paternalism as an obvious and natural part of life in nursing homes. Previous studies have highlighted the negative effects dependence and care routines can have on residents’ autonomy, freedom, and dignity because they conflict with the residents’ need to be in control of their own life.^2,26–28^ Loss of independence is considered a major threat to dignity and autonomy.^27,28^ Independence is regarded a major norm constituting dignity in our society, and dependence also influences the ability to uphold norms that contribute to personal and social dignity,^11^ like standards of hygiene and appearance, which uphold respect, belonging and esteem.

It was important for residents to feel included and to have the possibility to influence on decisions even though many decisions were assigned to caregivers. Even when residents agreed with both the reasoning and the result of the decision, residents felt violated when they were left out of major decisions. Residents would acknowledge a diminishing capacity for independence and a need of support in making decisions but being left out of the discussion entirely made them feel underestimated, excluded, and violated. In addition, it made the residents feel that they were not granted the proper appraisal respect.^6, 11^ Being left out violated their dignity by not confirming them as persons^14^ with rights to participate in decisions concerning themselves, and by underestimating their abilities to understand situations. Paternalism harms both autonomy and dignity by disrespecting someone’s right to be autonomous and their capacity for autonomy.^6^ Being excluded and underestimated felt very debasing to the residents, even though residents agreed with both the reasoning and the final decision that was made.

Residents in our study surrendered to dependency as they were not able to be independent anymore. This included being dependent on help for decisions. Residents experienced that they had lost motivation to do activities they knew they needed, and thus were dependent on pressure from caregivers to be able to perform these activities. Pressure as well as routines and regulations in the nursing home are usually considered paternalistic limitations of autonomy.^3^ But, the residents experienced that they needed them to be able to live according to their own standards. Soft paternalism presupposes a mismatch between peoples actions and their authentic will, due to factors like lack of willpower and cognitive bias.^6,19^ The dependence on pressure, routines and regulations that the residents experienced, can be understood as residents experiencing this mismatch between desires in the moment, and their authentic will due to a lack of motivation. Therefore, the residents regarded the paternalistic influence as assistance, helping them to act in accordance with whom they truly are, and not in violation of their dignity.

When residents experienced that the adherence to norms, such as regulations and routines were limiting their personal freedom, residents seemed to accept this as a natural compromise. Though routines that make residents wait for help harm residents experience of dignity,^29^ the residents regarded some limitation of personal freedom as a natural trade-off to get necessary help, and as adjustment to a new environment.^30^ Residents expressed that because they regarded the nursing home as a pre-existing social entity that they joined, it was natural to adhere to the existing nursing home norms. In addition, the residents seemed to consider the nursing home’s regulations, routines, and norms as necessary and reasonable, therefore it was obvious to them that they should adhere to the norms. The residents did not feel that they lost a right to autonomy or that their capacity was underestimated by adhering to the norms,^6^ since they were equal to all. This meant that it was only when residents felt that norms, regulations, and routines were unreasonable or disrespected their needs that they were experienced as harming dignity.

The professional caregivers were the ones who had knowledge, experience, and expertise in how to assist with care, and health issues, therefore it was obvious to residents that caregivers should take the lead in the nursing home. Residents were reluctant to give control to the caregivers when they experienced caregivers having insufficient knowledge and understanding. This corresponds with the view that caregivers have the role as leaders in the relationship between residents and caregivers.^21^ From this perspective, caregivers have the responsibility to lead residents toward a set of shared goals. To do this, the caregivers must align their expertise pertaining to health, illness, care, and context with the residents knowledge of themselves.^21^ Thus, the caregivers must know the resident as an individual,^7,14^ understand their situation and listen to them to be able to include the residents perspective. Overall, it presupposes that caregivers includes residents in major decisions, even though residents entrust caregivers with a certain decisional responsibility.

The decision to let caregivers take the lead in the relationship was also influenced by residents need to be regarded as ‘good residents’. The nursing home norms prescribes residents to accept the situation, to be modest, appreciative, and grateful for receiving help. Upholding these norms made residents feel like they were ‘good residents’ and bestowed them with esteem and social standing. Furthermore, residents risk condemnation and informal sanctions by other residents and caregivers if they do not uphold these norms. This means that these norms are dignitarian norms.^11^ Thus, the residents weighted their need of control, and of making requests, against the need for personal and social dignity that came from upholding these norms. Residents’ tendency to surrender to caregivers control can also be interpreted as a sign of residents not wanting to challenge caregivers and stand up for themselves because they are dependent, and fear punishment.^28^ However, residents also gain self-respect, a sense of dignity^29^ and social standing among peers, when they appear to accept their situation of dependence, are grateful for receiving help, and accept caregivers leadership. Residents accepting their situation of dependency and the nursing home norms can therefore be interpreted as residents replacing the unattainable ideal of independence, with dignitarian norms that the residents are still able to uphold.

Disregarding an explicit wish is usually considered harmful to both autonomy and dignity.^6^ However, the residents described situations where they seemed to appreciate that caregivers did not respect their explicit wishes. One resident even praised the caregivers for not listening to her refusal, saying: ‘*That there is someone who does not give up until I really must give up, that is important’.* This was surprising as the resident had previously emphasized the importance of having the possibility to make wishes and to ‘say no’. In those situations where the disregard of expressed wishes was appreciated, the residents experienced the paternalistic influence as a validation, and a sign of appraisal respect.^6^ This indicated that the caregiver considered them strong, confident, and capable enough to carry on with the activity and resist pressure if necessary. These situations are soft paternalistic in regard to being situations where the residents expressed desires in the moment that did not line up with their authentic values and goals,^6^ and the paternalistic influence was resistible.^19^ However, when caregivers use autonomy as justification for failing to provide residents with necessary care,^3^ when caregivers are overly attentive in respecting residents expressed wishes, the residents do not get the care they require. In addition, the residents might perceive this overprotection of their autonomy as an underestimation of their capabilities and strength to withstand pressure, and thus a violation of their dignity.^11^ This means that residents experience being subject to soft paternalism as dignifying, if the paternalistic influence is resistible, implying a positive appraisal of the their competency, and helps them pursue their authentic goals and values.

## Limitations and strengths of the study

The interviews for this study were carried out while there were strict COVID-19 restrictions in nursing homes. These restrictions meant that residents’ families and relatives had limited access to visit the nursing home, and the interviewer had to wear a mask, in some cases also a face shield, during the interviews. Residents were not wearing masks.

Restrictions on relatives’ access to the nursing home influenced the recruitment of participants. It was difficult to obtain consent from the resident’s next of kin during periods when relatives were not allowed to visit the nursing home. Recruitment of residents, requiring consent from both resident and guardian, thus became difficult. Sample variation was still ensured through active purposive sampling and an extension of the timeline.

The covering of the interviewer’s face was a limitation, as it negatively affected the quality of communication with residents. The residents were not able to see the interviewer’s face. This limitation was reduced by the interviewer carrying out participant observation in the nursing home before the interviews, enabling the residents and interviewer to become familiar with each other. This also allowed for residents to get used to the interviewer wearing a face mask during communication. Participant observation at the nursing home also strengthened the quality of the interviews as it gave the interviewer and residents a common frame of reference. This enabled the interviewer to ask better questions and was also a strength during the interpretation.

## Conclusions

Our findings indicate that residents submit to caregivers, giving them responsibility and leadership functions in the relationship. Moreover, residents experience paternalism as dignifying in situations where such influence is resistible and contributes to living according to their authentic goals and values. It is also dignifying when the paternalistic influence implies respect and appraisal of their capabilities. However, paternalism is experienced as debasing and harmful when residents feel excluded, or underestimated. This includes underestimation of their capability to withstand paternalistic influence.

This new understanding of how residents experience paternalism will be valuable to caregivers who experience dilemmas between autonomy and beneficence. Furthermore, it might help resolve situations where respect for residents’ autonomy creates failure to provide the necessary care. Our findings indicate that caregivers in nursing homes may use soft paternalism without violating residents’ dignity, if residents are allowed to influence decisions and situations involving their interests, and the caregiver conveys respect for their remaining competence. However, this understanding of paternalistic influences, involving caregivers’ respect might be specific to these particular residents in this context. Variations and nuances likely exist. Hence, further research in different contexts is required.
